# Statistical models and computational tools for predicting complex traits and diseases

**DOI:** 10.5808/gi.21053

**Published:** 2021-12-31

**Authors:** Wonil Chung

**Affiliations:** 1 Department of Statistics and Actuarial Science, Soongsil University, Seoul 06978, Korea; 2 Program in Genetic Epidemiology and Statistical Genetics, Harvard T.H. Chan School of Public Health, Boston, MA 02115, USA

**Keywords:** computational tools, polygenic risk score, PRS models

## Abstract

Predicting individual traits and diseases from genetic variants is critical to fulfilling the promise of personalized medicine. The genetic variants from genome-wide association studies (GWAS), including variants well below GWAS significance, can be aggregated into highly significant predictions across a wide range of complex traits and diseases. The recent arrival of large-sample public biobanks enables highly accurate polygenic predictions based on genetic variants across the whole genome. Various statistical methodologies and diverse computational tools have been introduced and developed to compute the polygenic risk score (PRS) more accurately. However, many researchers utilize PRS tools without a thorough understanding of the underlying model and how to specify the parameters for the best performance. It is advantageous to study the statistical models implemented in computational tools for PRS estimation and the formulas of parameters to be specified. Here, we review a variety of recent statistical methodologies and computational tools for PRS computation.

## Introduction

Accurately predicting complex traits and diseases (e.g., type 2 diabetes, cancer, and asthma) based on an individual’s genetic variants is crucial for effective disease prevention and personalized treatment [1-8]. The genetic architecture of many diseases contains a substantial polygenic component, meaning that thousands of variants with small effects contribute to disease risk. This limited the predictive ability of genetic variants in early studies based on significant associations from genome-wide association studies (GWAS). However, genetic variants—mostly single-nucleotide polymorphisms (SNPs)—from GWAS, including variants well below genome-wide significance, can be aggregated into highly significant predictions of phenotypes across a wide range of complex traits and diseases. With the recent arrival of public biobanks with 500K‒1M samples, highly accurate polygenic prediction is poised to become a reality [9]. The emergence of highly accurate polygenic prediction has led to the revitalization of the polygenic risk score (PRS), which is the score for predicting a trait and disease risk, calculated as the weighted sum of risk alleles with predicted weights computed by coefficients from GWAS.

For accurate PRS estimation, various statistical methodologies have been proposed and diverse computational tools have been developed, such as PLINK (https://zzz.bwh.harvard.edu/plink/) [10], GCTA (Genome-wide Complex Trait Analysis, https://cnsgenomics.com/software/gcta/) [11], and LDpred (https://github.com/bvilhjal/ldpred) [12]. These PRS tools have been widely adopted for genetic risk prediction in practice due to their easy usability with a proper theoretical basis. These tools compute the PRS using various data types, including the individual-level genotype as well as summary-level data on the basis of multiple regression, linear mixed models (LMMs), and Bayesian models. Despite the popularity of PRS tools, many researchers have utilized them without a thorough understanding of the underlying model and how to specify the parameters for the best performance. To achieve better prediction, it is advantageous to study the statistical models implemented in computational tools for PRS estimation and the mathematical formula of parameters to be specified. A deep understanding of the underlying statistical models in PRS software and a comparison of their advantages and disadvantages will help researchers to find an appropriate PRS tool for themselves.

Here, we review various statistical methodologies and computational tools for PRS computation. First, we review summary-based PRS methods with a few published SNPs or whole SNPs from large-sample GWAS using PLINK [10] and LDpred [12], with two main considerations: non-infinitesimal genetic architecture and the linkage disequilibrium (LD) structure of the genotype data. Second, we review traditional best linear unbiased prediction (BLUP)-based prediction with individual-level genotypes using genomic BLUP (GBLUP) [13] and summary-level data using summary statistics BLUP (SBLUP) [14]. Third, we review Bayesian multiple regression (BMR)-based prediction with individual-level data using BayesR [15,16] and summary-level data using summary statistics BayesR (SBayesR) [17]. Fourth, we review penalized regression-based approaches using the least absolute shrinkage and selection operator (lasso) [18,19], the elastic net [20], and lassosum (https://github.com/tshmak/lassosum/) [21]. Fifth, we review statistical methods for jointly analyzing multiple phenotypes to further improve prediction accuracy using multi-trait GBLUP (MTGBLUP, https://github.com/uqrmaie1/mtgblup) [16], weighted multi-trait SBLUP (wMT-SBLUP, https://github.com/uqrmaie1/smtpred) [22], and cross-trait penalized regression (CTPR, http://lianglab.rc.fas.harvard.edu/CTPR/) [23]. Finally, we review multi-ethnic approaches to incorporate information from multiple populations using XP-BLUP (https://github.com/tanglab/XP-BLUP) [24], multi-ethnic PRS [25], and multi-ancestry PRS [26]. We conclude with a discussion of statistical models and computational tools that require further work on improving the accuracy of PRS prediction. Table 1 presents a list of the PRS methods reviewed in this paper, along with their underlying statistical models, computational tools, and required data.

## Polygenic Risk Prediction

A study of schizophrenia showed that the PRS achieved significantly better prediction in validation samples than random scores, and far more accurate than those based on the single GWAS loci discovered in the study [27]. This study describes an early demonstration of the importance and advantages of the PRS for the prediction of disease risk [28].

### Use of a few published SNPs

Let ${\hat{\beta }}_{j}$ denote the effect size for the published SNP *j* (i.e., a GWAS-significant SNP from previous GWAS studies), *x_ij_* denote the genotype for SNP *j* of individual *i*, and *y_i_* denote the phenotype of individual *i*. The predicted phenotype for individual *i* can be simply computed as ${\hat{y}}_{l}=\sum_{j}^{}{\hat{\beta }}_{j}x_{ij}$, which is defined as the PRS. For a continuous trait (e.g., height, weight, and body mass index [BMI]), the PRS is evaluated by the prediction *R*^2^, which is defined as the square of the correlation between the true and predicted phenotypic values. For a case-control trait (e.g., type 2 diabetes or cancer), the PRS is evaluated by the area under the curve (AUC) [29], pseudo-*R*^2^, and *R*^2^ on the liability scale [30]. The prediction *R*^2^ is bounded by the heritability explained by GWAS-significant SNPs ($h_{GWAS}^{2}$), the maximum proportion of phenotypic variance explained by a linear combination of those SNPs, because $h_{GWAS}^{2}$ is the theoretical limit of prediction *R*^2^ with GWAS-significant SNPs [31,32].

The prediction *R*^2^ of published SNPs depends on the genetic architecture of the phenotypes. Under an infinitesimal genetic architecture, all SNPs are causal with relatively small effect size, and thus the associated SNPs identified by GWAS studies explain a small amount of genetic variance, achieving poor prediction *R*^2^. For example, the narrow-sense heritability (*h*^2^) for BMI is *h*^2^=0.4-0.6, but the heritability explained by GWAS-significant SNPs with >300K samples yields $h_{GWAS}^{2}$=0.027, meaning a prediction *R*^2^ of 0.027 at most [2,33]. Instead, under a non-infinitesimal genetic architecture, only a subset of SNPs has moderate to large effects whereas most SNPs have zero effects; thus, the associated SNPs identified by GWAS studies explain more genetic variance, yielding higher prediction *R*^2^. For example, the narrow-sense heritability of type 1 diabetes is estimated as *h*^2^=0.9, but the heritability explained by GWAS-significant SNPs is $h_{GWAS}^{2}$=0.6, meaning that the published SNPs will achieve a prediction *R*^2^ of 0.6 at most [2,34].

### Use of all SNPs from GWAS studies

Polygenic risk prediction can be performed using all SNPs from GWAS studies, not only GWAS-significant SNPs. The PRS can be estimated as ${\hat{y}}_{l}=\sum_{j}^{}{\hat{\beta }}_{j}x_{ij}$ which utilizes both GWAS-significant and non-significant SNPs. That is, the SNPs are not required to be GWAS-significant. The prediction *R*^2^ is bounded by the heritability explained by the genotyped SNPs ($h_{g}^{2}$), the maximum proportion of phenotypic variance explained by a linear combination of genotyped SNPs. This is explained by the fact that the expected value of prediction *R*^2^ is $E[R^{2}]\approx h_{g}^{2}/[1+M/h_{g}^{2}N]$, where *M* is the total number of SNPs and *N* is the number of individuals [31,32]. Thus, $h_{g}^{2}$ is the theoretical limit of polygenic prediction in large-scale GWAS studies.

In order to utilize all SNPs to compute the PRS, there are two main considerations: (1) the non-infinitesimal genetic architecture of the phenotype, and (2) the LD structure of the genotype data. That is, ${\hat{y}}_{l}=\sum_{j}^{}{\hat{\beta }}_{j}x_{ij}$ does not account for a non-infinitesimal genetic architecture and LD structure since all SNPs are utilized to compute the PRS and those SNPs are assumed to be independent in the model. The standard heuristic approach for a non-infinitesimal architecture is p-value thresholding (P < P_T_), which only considers SNPs with a p-value (P) less than the threshold (P_T_). The best P_T_ threshold is selected when the threshold achieves the best prediction accuracy in validation samples. In the absence of an independent validation sample, the data can be divided into training and validation data sets, and threshold selection process is repeated with different partitions of the samples by performing k-fold cross-validation. The standard heuristic approach to account for LD structure is LD pruning and LD clumping. LD pruning randomly removes one of each pair of linked SNPs based on the genotypic correlation (r^2^), while LD clumping removes SNPs with less significant p-values for the phenotype among pairs of linked SNPs. Both pruning approaches also require optimization of the best r^2^ threshold in validation samples. P-value thresholding and LD-pruning are widely used for PRS computation, but these approaches do not achieve maximum prediction accuracy.

### PRS tools

Popular genetic tools, such as PLINK [10], PRSice (https://www.prsice.info/) [35], and PRSice-2 [36], are utilized to estimate PRS with a few published SNPs or all SNPs from GWAS studies. The PLINK is not originally designed for PRS computation, but every required procedure of the C+T (LD clumping + p-value thresholding) approach can be performed with PLINK. It requires the summary statistics from GWAS studies as well as phenotype, covariate, and genotype data from target samples after a quality control procedure. To account for the LD structure, LD clumping is performed using the PLINK options (e.g., *--clump-r2 0.1 --clump-kb 250*) to form clumps of all SNPs that are within a certain distance (in kilobases [kb]) from the index SNPs (e.g., 250 kb) and that are in LD with the index SNP based on the r^2^ threshold (i.e., r^2^ < 0.1). For p-value thresholding, the SNPs are generated with p-values less than a provided threshold (P_T_) and then candidate PRSs corresponding to the thresholds are created with the PLINK options (e.g., *--score, --q-score*). The best PRS is selected among candidate PRSs computed at a range of p-value thresholds based on the prediction *R*^2^. For the automation of the C+T approach in PLINK, we can utilize PRSice and PRSice-2, which are options in R software for computing and evaluating the PRS. PRSice and PRSice-2 are popular PRS tools and constitute efficient and scalable software for automating and simplifying PRS computation on large-scale GWAS data. They handle imputed data as well as genotyped data and simultaneously evaluate a large number of continuous and binary phenotypes. Similar to PLINK, they require summary data as well as phenotype, covariate, and genotype data for the target samples. They automate the procedure of the standard C+T method, which utilizes PLINK options for PRS analysis.

## LD-Based Prediction

A critical issue in estimating the PRS is the LD structure between SNPs, which has been heuristically addressed by LD pruning and LD clumping. Recently, LDpred was developed as a more sophisticated method that also utilizes summary statistics [12]. It has been shown that modeling LD using an LD reference panel and estimating the posterior mean of effect size can improve prediction accuracy [28].

### LDpred

LDpred is an LD matrix and summary statistics–based Bayesian method for polygenic prediction, which is a popular tool for deriving the PRS [12]. It computes posterior means under a point-normal prior, accounting for LD information. The PRS is computed by ${\hat{y}}_{l}=\sum_{j}^{}E(\beta_{j}|{\hat{\beta }}_{j})x_{ij}$, where ${\hat{y}}_{l}$ is the predicted phenotype for sample *i*, *β_j_* is the effect size for SNP *j*, *x_ij_* is the genotype for sample *i* and SNP *j*, and $E(\beta_{j}|{\hat{\beta }}_{j})$ is the posterior mean effect size for SNP *j*.

In the special case of no LD between SNPs, the posterior mean can be computed analytically. Under a Gaussian infinitesimal prior, $\beta_{j}\sim N(0,\frac{h_{g}^{2}}{M})$, the posterior mean effect size is derived as $E(\beta_{j}|{\hat{\beta }}_{j})=\frac{h_{g}^{2}}{h_{g}^{2}+M/N}{\hat{\beta }}_{j}$, where ${\hat{\beta }}_{j}\sim \beta_{j}+\epsilon_{j},\;\epsilon_{j}\sim N(0,\;\frac{1}{N})$, which can be interpreted as uniform shrinking of the estimated effect size for SNP *j*, ${\hat{\beta }}_{j}$. Under a Gaussian non-infinitesimal prior, $\beta_{j}\sim N\;(0,\;\frac{h_{g}^{2}}{Mp})$ with probability p, and *β_j_*~0 with probability 1-p, where p is the proportion of causal SNPs. The posterior mean effect size is estimated as $E(\beta_{j}|{\hat{\beta }}_{j})=\frac{h_{g}^{2}}{h_{g}^{2}+Mp/N}{\bar{p}}_{j}{\hat{\beta }}_{j}$ where ${\bar{p}}_{j}$ is posterior probability that the *j*th SNP is causal, which can be interpreted as non-uniform shrinking of the estimated effect size ${\hat{\beta }}_{j}$.

In the case of LD between SNPs, the posterior means can be computed analytically only with an infinitesimal prior. Under a Gaussian infinitesimal prior, the posterior mean effect size is derived as $E(\beta_{j}|{\hat{\beta }}_{j})={\left[D+\frac{M}{Nh_{g}^{2}}I\right]}^{-1}\;{\hat{\beta }}_{j}$ where *D* is an LD matrix (*M*×*M*) that needs to be estimated by the LD in a reference panel (LDpred-inf). LDpred-inf is a natural extension of the GBLUP to summary statistics. Under a Gaussian non-infinitesimal prior, posterior means cannot be computed analytically but they can be computed with Markov-chain Monte Carlo Gibbs samplers. First, *β_j_* values are initialized based on an infinitesimal prior with LD (LDpred-inf). At each iteration, *β_j_* is resampled from ${\hat{\beta }}_{j}\sim N(D\beta ,\;D/N),\;f(\beta_{j}|{\hat{\beta }}_{j})=f(\beta_{j})e^{-\frac{N}{2}(\hat{\beta }-D\beta {)}^{T}D^{-1}(\hat{\beta }-D\beta )}$, where *f*(*β_j_*) reflects the point-normal prior (based on $h_{g}^{2}$ and p). Generally, 100 big iterations suffice for convergence, and the posterior means are averaged to estimate ${\hat{\beta }}_{j}$. The PRS is computed based on the estimated posterior means of the SNP effects and genotype data from the target dataset.

### LDpred software

The procedure for computing the PRS using LDpred consists of three steps: (1) synchronizing the genotype and summary data, (2) generating LDpred SNP weights, and (3) generating the individual PRS. The first step synchronizes genotype and summary statistics and then generates the coordinated genotype data with the ‘*ldpred coord*’ command. It requires one genotype file (LD reference) with at least 1,000 individuals of the same ancestry as the individuals for summary statistics. The second step generates an LD information file with a pre-specified LD radius and re-weights the SNP effects with the ‘*ldpred gibbs*’ command. One LD information file is created with a pre-specified LD radius, but several SNP weight files are generated corresponding to the different values of p (the proportion of causal SNPs). The third step computes the PRS for individuals in the target dataset with the ‘*ldpred score*’ command. Separate PRS files are generated corresponding to the different values of p. Additionally, LDpred provides a pruning and thresholding option as an alternative method with the ‘*ldpred p*+*t*’ command. This option often yields better prediction results than the original LDpred when the sample size of LD reference panel is not large enough.

The construction of a genome-wide PRS using LDpred requires summary statistics from existing large-scale GWAS studies (e.g., the UK Biobank [37-39], DIAGRAM [40]) and an LD reference panel (e.g., the 1000 Genomes project) [41]. A set of candidate PRSs is computed with ranging causal fractions ranging from 0.001 to 1 with p-value thresholding and LD pruning. A range of p-values and pairwise correlations in the LD reference panel are used to include the significantly-associated SNPs for each LD-based clump across the genome with various thresholds [9]. The candidate PRSs are calculated in a validation dataset by multiplying the genotype dosage for each variant by its corresponding weight and summing across all SNPs. The optimal model is selected based on the maximal AUC computed in a validation dataset, and the PRS in the target dataset is then computed. The association between the computed PRS and the target traits is evaluated using linear regression (for a continuous trait) or logistic regression (for a binary trait) with adjustment for covariates (e.g., age, sex, and genotype principal components). The inclusion of such covariates generally leads to more accurate estimates of the PRS and increases the prediction accuracy, but makes it difficult to quantify the exact genetic effects on the target trait. Thus, reporting PRS results with and without important covariates is recommended.

Recently, LDpred-2, a new version of LDpred, was developed to improve predictive performance compared to LDpred [42]. It provides two new options: (1) the ‘*sparse*’ option, which can make SNP effects exactly 0; and (2) the ‘*auto*’ option, which learns the tuning parameter *p*, which is the proportion of causal SNPs, directly from the dataset. LDpred-2 was implemented in the R package ‘*bigsnp*’.

## BLUP-Based Prediction

An alternative to summary-based approaches is to fit the effect sizes of all SNPs simultaneously using BLUP models, which is a more traditional approach for computing the PRS. Fitting all SNPs simultaneously is more appropriate than summary-based approaches, producing more accurate predictors.

### GBLUP

GBLUP methods utilize individual-level GWAS data, not summary statistics, to estimate SNP effects using LMMs. The GBLUP model is *y*=*Xβ*+*g*+*e*, where *y* is a vector of phenotypes (*N*×1), *X* is a matrix of covariates excluding the SNPs (*N*×*C*), *β* is a vector of covariate effects (*C*×1) and *g* is a vector of random genetic effects for all individuals with $g\sim N(0,\;\sigma_{g}^{2}A)$ (*N*×1) (*A* is a *N*×*N* genetic related matrix [GRM]) and *e* is a vector of random errors with $e\sim N(0,\;\sigma_{e}^{2}I)$ (*N*×1). The genetic values (i.e., individual BLUP) are estimated as $\hat{g}=E(g|y)=\sigma_{g}^{2}A(\sigma_{g}^{2}A+\sigma_{e}^{2}I{)}^{-1}\;(y-X\beta )$, requiring the computation of the inverse of *N*×*N* matrix. A GBLUP model can be transformed to a ridge regression BLUP model (RRBLUP) [43,44], which is *y*=*Xβ*+*Wu*+*e*, where *W* is a matrix of standardized genotypes (*N*×*M*) and *u* is a vector of random SNP effects with $u\sim N(0,\;\sigma_{u}^{2}I)$ (*M*×1). The SNP effects (i.e., SNP BLUP) are estimated as $\hat{u}=E(u|\hat{g})=W^{T}A^{-1}\hat{g}/M$, requiring GRM *A* and individual BLUP $\hat{g}$ from GBLUP models. The individual BLUP in target samples is computed as ${\hat{g}}_{new}=W_{new}\hat{u}$, where *W_new_* is a matrix of standardized genotypes in the target dataset, $\hat{u}$ is a vector of SNP effects computed from the training dataset, and ${\hat{g}}_{new}$ is considered as the PRS for the target dataset.

### SBLUP

The GBLUP models require individual-level genotype and phenotype data for training, but this is not always possible. Instead, summary SBLUP models can be utilized by approximating individual-level genotype and phenotype data using summary statistics and a reference panel [14]. The SBLUP model is similar to the LDpred model, but it only considers the infinitesimal case, which corresponds to the LDpred-Inf model. The SNP effects (i.e., SNP BLUP) in the RRBLUP model are re-written as $\hat{u}=(W^{T}W+\lambda I{)}^{-1}W^{T}y$ with $\lambda =\frac{\sigma_{e}^{2}}{\sigma_{u}^{2}}$. The SBLUP model approximates the covariance matrix of genotypes in the training data by genotype data from a reference panel as $E(W^{T}W)=V^{T}V*(\frac{n_{t}}{n_{r}})=B$, where *V* is a matrix of standardized genotypes from the reference panel, and *n_t_* and *n_r_* are the sample sizes for the training and reference samples, respectively. This assumes the similarity of allele frequencies and LD structure between training and reference samples. It also approximates $E(W^{T}y)=diag(B)\hat{\beta }$, where $\hat{\beta }$ is the least square estimate (LSE) for SNP effects, which are estimated using summary statistics. The SNP effects in the RR BLUP are finally written as $\hat{u}=(B+\lambda I{)}^{-1}diag(B)\hat{\beta }$, where $B=V^{T}V*(\frac{n_{t}}{n_{r}})$. The heritability is computed as $h_{g}^{2}=\frac{M\sigma_{u}^{2}}{\sigma_{p}^{2}}$, where $\sigma_{p}^{2}$ is the phenotypic variance (~1 due to standardization of the genotype data in the training data); thus, we have $\lambda =M(\frac{1}{h_{g}^{2}}-1)$. The individual BLUPs in the target samples are computed as ${\hat{g}}_{new}=W_{new}\hat{u}$, which is the same as those in the GBLUP models.

### GCTA software

GCTA software was initially designed to estimate SNP-based heritability and has been extended for many other genetic analyses including GBLUP and SBLUP. For GBLUP analysis, the GRM (*A*) is first estimated from the training genotype data with the ‘*--make-grm*’ option, and then the individual BLUP ($\hat{g}$) is computed from the estimated GRM and the training phenotype data with the ‘*--reml-pred-rand*’ option. The SNP BLUP ($\hat{u}$) is transformed from the output of the individual BLUP ($\hat{g}$) with the ‘*--blup-snp*’ option and used to predict the PRS of individuals in independent validation data with the PLINK option ‘*--score*’. For SBLUP analysis, the SNP BLUP ($\hat{u}$) is computed from the GWAS summary data and LD reference data, as well as the pre-specified input parameter (λ) with the ‘*--bfile*’, ‘*--cojo-file*’ and ‘*--cojo-sblup*’ options. The PRS of individuals in validation data is computed using PLINK, which is the same as in GBLUP models.

## BMR-Based Prediction

BMR methods extend the standard LMM by including an alternative prior for SNP effects, further improving prediction accuracy [14,15,17].

### BayesR

The BMR model, BayesR [15,16] assumes that the phenotype is related to set of SNPs under a multiple linear regression model: *y*=*Xβ*+*e* where *y* is a vector of centered phenotypes (*N*×1), *X* is a matrix of standardized genotypes (*N*×*M*), *β* is a vector of SNP effects (*M*×1) and *e* is a vector of random errors with $e\sim N(0,\;\sigma_{e}^{2}I)$ (*N*×1). It also assumes the SNP effects result from a finite normal mixture of *C* components, so that the prior for *β* becomes $P(\beta_{j}|\pi ,\;\sigma_{\beta }^{2})=\sum_{c=1}^{C}\pi_{c}N(\beta_{j}|0,\;\sigma_{\beta c}^{2})$, where $N(\beta_{j}|0,\;\sigma_{\beta c}^{2})$ denotes the normal density with mean 0 and variance $\sigma_{\beta c}^{2}$ and *π*=(*π*_1_,…,*π_C_*) and $\sigma_{\beta }^{2}=(\sigma_{\beta 1}^{2},\;...,\;\sigma_{\beta C}^{2})$. The posterior for *β* is $P(\beta |\pi ,\;\sigma_{\beta }^{2},\;\sigma_{e}^{2})\;\propto \;P(\beta |\pi ,\;\sigma_{\beta }^{2})P(\pi )P(\sigma_{\beta }^{2})P(\sigma_{e}^{2})$ and *β* is sampled using the Gibbs sampling scheme. The posterior mean for SNP effects ($E(\beta |\pi ,\;\sigma_{\beta }^{2},\;\sigma_{e}^{2})$) from the BayesR method is used as the estimated SNP effect, and the PRS of validation samples is computed using the estimated SNP effects and validation genotype data.

### SBayesR

The BayesR model with individual-level data was extended to utilize summary statistics from GWAS studies in SBayesR [17]. The SBayesR model relates estimates of multiple regression coefficients (*β*) to estimates of regression coefficients from *M* simple linear regression (*b*) by multiplying *y*=*Xβ*+*e* by *D*^-1^*X^T^*, where $D=\mathrm{diag}(x_{1}^{T}x_{1},\;...,\;x_{M}^{T}x_{M})$ to result in (*D*^-1^*X^T^*)*y*=(*D*^-1^*X^T^*)*Xβ*+(*D*^-1^*X^T^*)*e*. Noting that *b*=*D*^-1^*X^T^y* is the vector of the least squares marginal regression effects estimates and $B=D^{-\frac{1}{2}}X^{T}XD^{-\frac{1}{2}}$ is the LD correlation matrix between all SNPs, the multiple regression model is re-written as $b=D^{-\frac{1}{2}}BD^{-\frac{1}{2}}\beta +D^{-1}X^{T}e$ and the following likelihood can be proposed for multiple regression coefficients $\left(\beta ):\;L(\beta ;\;b,\;D,\;B):=N(b;\;D^{-\frac{1}{2}}BD^{\frac{1}{2}}\beta ,\;D^{-\frac{1}{2}}BD^{-\frac{1}{2}}\sigma_{e}^{2}\right)$. Due to the unavailability of individual-level data, *D* is replaced by the estimates $\hat{D}=diag(N_{1},\;...,\;N_{M})$ thanks to the standardized SNPs and *B* is replaced by $\hat{\beta }$, an estimate computed from a reference sample of the same ancestry as the samples for GWAS summary statistics. We assume that the prior for *β* is $P(\beta_{j}|\pi ,\;\sigma_{\beta }^{2})\;=\;\sum_{c=1}^{C}\pi_{c}N(\beta_{j}|0,\;\gamma_{C}\sigma_{\beta }^{2})$, where *C* denotes the pre-specified maximum number of components in the finite mixture model, *π*=(*π*_1_,…,*π_C_*) and *γ*=(*γ*_1_,…,*γ_C_*). The default values are *C*=4, *γ*=(0,0.01,0.1,1). The posterior for *β* is $P(\beta |b,\;D,\;B,\;\pi ,\;\sigma_{\beta }^{2},\;\sigma_{e}^{2})\;\propto \;P\left(\beta |D,\;B,\;\sigma_{\beta }^{2},\;\sigma_{e}^{2}\right)P(b|\beta ,\;D,\;B)P(\pi )$ $P(\sigma_{\beta }^{2})P\left(\sigma_{e}^{2}\right)$. The coefficients, *β*, are sampled using the Gibbs sampling approach and the posterior mean for the SNP effects ($E(\beta |b,\;D,\;B,\;\pi ,\;\sigma_{\beta }^{2},\;\sigma_{e}^{2})$) from the SBayesR method is used as the estimated SNP effects.

### GCTB software

GCTB (Genome-wide Complex Trait Bayesian Analysis, https://cnsgenomics.com/software/gctb/) is a software tool that contains a family of Bayesian LMMs for complex trait analyses using GWAS SNPs. First of all, GCTB specifies the Bayesian alphabet for the analysis with the option ‘*--bayes*': R for BayesR. The options ‘*--pi 0.05*’ (a starting value for sampling *π*) and ‘*--hsq 0.5*’ (a starting value for sampling $\sigma_{\beta }^{2}$ and $\sigma_{e}^{2}$ on the basis of SNP-based heritability) need to be specified. Second, GCTB specifies the summary Bayesian alphabet for the analysis with the option ‘*--sbayes*’: R for SbayesR. The full chromosome-wide LD matrices are estimated using multiple CPUs with the ‘*--make-full-ldm*’ option, and shrunk LD matrices are built with the ‘*--make-shrunk-ldm*’ option. SBayesR models are conducted with the options ‘*--pi 0.95, 0.02, 0.02, 0.01*', ‘*--ldm*’ (an LD matrix), ‘*--gamma 0,0.01,0.1,1*’ (a prespecified hyperparameter *γ*), and ‘*--gwas-summary*’ (an input file for GWAS summary statistics).

## Penalized Regression-Based Prediction

GBLUP-based methods implicitly assume an infinitesimal genetic architecture, whereas in reality complex traits or diseases are estimated to have roughly only a few thousand causal SNPs in the genome [45,46]. This fact has provided motivation for efforts to construct a PRS that accommodates a non-infinitesimal genetic architecture using penalized regression-based prediction methods.

### Lasso and elastic net

Penalized regression methods such as the lasso [18,19], the elastic net [20], the adaptive lasso [47], or other statistical learning methods [48] have previously been evaluated for genomic risk prediction [49,50]. The traditional linear regression model is *y*=*Xβ*+*e*, where *y* is a vector of phenotypic values (*N*×1), *X* is a matrix of genotypes (*N*×*M*), *β* is a vector of SNP effects (*M*×1) and *e* is random error with $e\sim N(0,\;\sigma_{e}^{2}I)$ (*N*×1). The elastic net regression obtains the estimates of *β* by minimizing the following object function: $f(\beta )=(y-X\beta {)}^{T}(y-X\beta )+\lambda \left[\alpha {\left|\left|\beta \right|\right|}_{1}+(1-\alpha {\left|\left|\beta \right|\right|}_{2}^{2}/2)\right]$, where ${\left|\left|\beta \right|\right|}_{1}=\sum_{j=1}^{M}\left|\beta_{j}\right|$ is the *L*_1_ norm of *β*, ${\left|\left|\beta \right|\right|}_{2}=\sqrt{\sum_{j=1}^{M}\beta_{j}^{2}}$ is the *L*_2_ norm of *β*, and *λ* and *α* are tuning parameters to be estimated. When *α*=1,*f*(*β*) becomes the object function for lasso regression, and when *α*=0, it becomes the object function for ridge regression. The PRSs in target samples are constructed with the estimated SNP effects from the lasso or elastic net and genotype data from the target dataset.

### Lassosum

The lassosum is a method for computing lasso or elastic net estimates using GWAS summary statistics and an LD reference panel [21]. The object function for lasso is given by $f(\beta )=(y-X\beta {)}^{T}(y-X\beta )+\lambda {\left|\left|\beta \right|\right|}_{1}=yy^{T}-2\beta^{T}X^{T}y+\beta^{T}X^{T}\;X\beta +\lambda {\left|\left|\beta \right|\right|}_{1}$, which is equivalent to $yy^{T}-2\beta^{T}r+\beta^{T}R\beta +\lambda {\left|\left|\beta \right|\right|}_{1}$, where *r*=*X^T^y* is the SNP-wise correlation between the SNPs and the phenotype and *R*=*X^T^X* is the LD matrix, a matrix of correlations between SNPs. The lassosum approximates *R* by $R=(1-s)X_{r}^{T}X_{r}+sI$ for some 0<*s*<1 where *X_r_* is matrix of genotypes from a reference panel and also approximates *r* by obtaining publicly available summary statistics. The lassosum constructs PRSs using summary statistics and a reference panel in a penalized regression setting.

### R packages

The most popular tool for lasso, ridge, and elastic net regression is ‘glmnet’ in R (https://cran.r-project.org/web/packages/glmnet/). The ‘glmnet’ package fits a generalized linear model via the penalized maximum likelihood approach. It is not originally designed for GWAS studies, but it is widely used for PRS analyses due to its computational efficiency. The lassosum is a R package or standalone software for performing lasso or elastic net regression with summary data and an LD reference panel. The reference panel is assumed to be in the PLINK format, and the GWAS summary statistics are given as data.frame in R.

## Multi-Trait Approaches

Recent studies have shown that GWAS of related phenotypes further improve the accuracy of polygenic predictions [23,25,51]. Human complex traits and disease traits share genetic architecture with other genetically related traits; therefore, the integration of multiple traits through appropriate methods would achieve improvement in prediction accuracy.

### MTGBLUP

In order to utilize multiple traits to improve prediction accuracy, the RRBLUP and GBLUP methods are extended to the bivariate ridge regression method [52] and MTGBLUP [13,16,43,44], which treat genetic effects as random to obtain individual BLUP and SNP BLUP using one or more genetically correlated traits. The GBLUP models are readily extended to multiple traits (*T* traits): *y_i_*=*X_i_β_i_*+*g_i_*+*e_i_*=*X_i_β_i_*+*W_i_u_i_*+*e_i_* (i.e. *g_i_*=*W_i_u_i_*) where $g_{i}\sim N(0,\;\sigma_{gi}^{2}A)$ and $e_{i}\sim N(0,\;\sigma_{ei}^{2}I)$ for *i*=1,…,*T*. The individual BLUP model $({\hat{g}}_{1},\;...,\;{\hat{g}}_{T}{)}^{T}$ and the SNP BLUP model $({\hat{u}}_{1},\;...,\;{\hat{u}}_{T}{)}^{T}$ for *T* traits are given as:

$\left[\begin{array}{c} {\hat{g}}_{1}\\ \vdots \\ {\hat{g}}_{T} \end{array}\right]=\left[\begin{array}{ccc} {\sigma_{g}}_{1}^{2} & \cdots & \sigma_{g1T}\\ \vdots & \ddots & \vdots \\ \sigma_{gT1} & \cdots & {\sigma_{g}}_{T}^{2} \end{array}\right]⊗A\cdot V^{-1}\left[\begin{array}{c} y_{1}-X_{1}\beta_{1}\\ \vdots \\ y_{T}-X_{T}\beta_{T} \end{array}\right]\;where\;V\;=\left[\begin{array}{ccc} A{\sigma_{g}}_{1}^{2}+I_{N}{\sigma_{e}}_{1}^{2} & \cdots & A\sigma_{g1T}+I_{N}\sigma_{e1T}\\ \vdots & \ddots & \vdots \\ A\sigma_{gT1}+I_{N}\sigma_{e1T} & \cdots & A{\sigma_{g}}_{T}^{2}+I_{N}{\sigma_{e}}_{T}^{2} \end{array}\right],\;\left[\begin{array}{c} {\hat{u}}_{1}\\ \vdots \\ {\hat{u}}_{T} \end{array}\right]={\left[\begin{array}{ccc} W_{1} & \cdots & 0\\ \vdots & \ddots & \vdots \\ 0 & \cdots & W_{T} \end{array}\right]}^{T}⊗A^{-1}\;\left[\begin{array}{c} {\hat{g}}_{1}\\ \vdots \\ {\hat{g}}_{T} \end{array}\right]\cdot M^{-1}={\left[\begin{array}{ccc} W_{1} & \cdots & 0\\ \vdots & \ddots & \vdots \\ 0 & \cdots & W_{T} \end{array}\right]}^{T}\left[\begin{array}{ccc} {\sigma_{g}}_{1}^{2} & \cdots & {\sigma_{g}}_{1T}^{1}\\ \vdots & \ddots & \vdots \\ {\sigma_{g}}_{T1}^{2} & \cdots & {\sigma_{g}}_{T}^{2} \end{array}\right]⊗I_{N}\cdot V^{-1}\left[\begin{array}{c} y_{1}-X_{1}\beta_{1}\\ \vdots \\ y_{T}-X_{T}\beta_{T} \end{array}\right]M^{-1}$

The individual BLUP in a validation sample $({\hat{g}}_{1,\;new},\;...,\;{\hat{g}}_{T,\;new}{)}^{T}$ can be computed as $\left[\begin{array}{c} {\hat{g}}_{1,new}\\ \vdots \\ {\hat{g}}_{T,new} \end{array}\right]=\left[\begin{array}{ccc} W_{1,new} & \cdots & 0\\ \vdots & \ddots & \vdots \\ 0 & \cdots & W_{T,new} \end{array}\right]\left[\begin{array}{c} {\hat{u}}_{1}\\ \vdots \\ {\hat{u}}_{T} \end{array}\right]$ = *W_new_* $\left[\begin{array}{c} {\hat{u}}_{1}\\ \vdots \\ {\hat{u}}_{T} \end{array}\right]$ where *W_new_* is a matrix of standardized genotypes in the target dataset and $({\hat{u}}_{1},\;...,\;{\hat{u}}_{T}{)}^{T}$ is a vector of SNP effects computed from the training dataset.

### wMT-SBLUP

The wMT-SBLUP [22] creates the PRS as a weighted index that combines published GWAS summary statistics across many different traits. The SNP BLUP for *T* traits can be re-written as $\left[\begin{array}{c} {\hat{u}}_{1}\\ \vdots \\ {\hat{u}}_{T} \end{array}\right]={\left[W^{T}W+\sum_{\epsilon }^{}\sum_{u}^{-1}⊗I_{M}\right]}^{-1}W^{T}y$, where $W=\left[\begin{array}{ccc} W_{1} & \cdots & 0\\ \vdots & \ddots & \vdots \\ 0 & \cdots & W_{T} \end{array}\right],\;\sum_{\epsilon }^{}=\left[\begin{array}{ccc} \sigma_{\epsilon 1}^{2} & \cdots & 0\\ \vdots & \ddots & \vdots \\ 0 & \cdots & \sigma_{\epsilon T}^{2} \end{array}\right],\;\sum_{u}^{}=\left[\begin{array}{ccc} \sigma_{g1}^{2} & \cdots & \sigma_{g1T}\\ \vdots & \ddots & \vdots \\ \sigma_{gT1} & \cdots & \sigma_{gT}^{2} \end{array}\right]$. Similar to SBLUP methods, $E(W_{i}^{T}W_{i})=N_{i}L$ and $E(W_{i}^{T}y_{i})=N_{i}{\hat{\beta }}_{i}$, where *L* is an *M*×*M* scaled LD correlation matrix estimated from a reference panel and ${\hat{\beta }}_{i}$ is the LSE for SNP effects, which are computed from GWAS summary statistics. The SNP BLUP for *T* traits can be approximately computed as $\left[\begin{array}{c} {\hat{u}}_{1}\\ \vdots \\ {\hat{u}}_{T} \end{array}\right]={\left[I_{k}⊗L+\sum_{\epsilon }^{}\sum_{u}^{-1}N^{-1}⊗I_{M}\right]}^{-1}\hat{\beta }$, where $\hat{\beta }=\left[\begin{array}{c} {\hat{\beta }}_{1}\\ \vdots \\ {\hat{\beta }}_{T} \end{array}\right],\;N=diag(N_{1},\cdots ,\;N_{T})$. The individual BLUP in target samples can be computed similarly to the process for MT-GBLUP.

### CTPR

To utilize multiple traits for PRS computation, the CTPR method was developed [23]. The SNP coefficients are estimated using the following equation: $\hat{\beta }=\begin{array}{c} argmin\;RSS(\beta )\\ \beta \end{array}+p_{\lambda_{1}}^{sp}(\beta )+p_{\lambda_{2}}^{ctp}(\beta )$, where *RSS*(*β*) is the residual sum of squares, $p_{\lambda_{1}}^{sp}(\beta )$ is sparsity penalty with a tuning parameter *λ*_1_ using lasso or the minimax concave penalty to induce a sparse solution, and $p_{\lambda_{2}}^{ctp}(\beta )$ is the cross-trait penalty with a tuning parameter *λ*_2_ to incorporate shared genetic effects across multiple traits for large-sample GWAS data. It induces smoothness of the coefficients and can incorporate prior knowledge on the similarity of a pair of traits at a given SNP via adjacency coefficients. It also incorporates multiple secondary traits based on individual-level genotypes and/or summary statistics. The PRS in target samples is computed as ${\hat{y}}_{t}=X_{t}\hat{\beta }$, where *X_t_* is a matrix of standardized genotypes in the target dataset, $\hat{\beta }$ is a vector of the estimated SNP effects from CTPR, and ${\hat{y}}_{t}$ is considered as the PRS for the target dataset.

## Multi-ethnic Approaches

Genetic risk prediction in diverse populations currently lags far behind risk prediction in European samples [25,53]. Striking examples include a reported relative decrease of 53%‒89% in schizophrenia risk prediction accuracy in Japanese and African-American populations [12] and 70%‒80% in BMI and type 2 diabetes prediction accuracy in those of African ancestry [54] compared to Europeans in studies using European training data due to between-population differences in population allele frequencies and patterns of LD. An alternative is to use training data from the target population, but this generally implies a much lower training sample size, reducing prediction accuracy. A recent method that incorporates training data from European and non-European populations improves prediction accuracy by using XP-BLUP [24] with the use of European-discovered SNPs and population-specific weights or by using a multi-ethnic PRS [25] and multi-ancestry PRS [26] with averages across all admixed individuals.

## Discussion

**Fig. 1.**
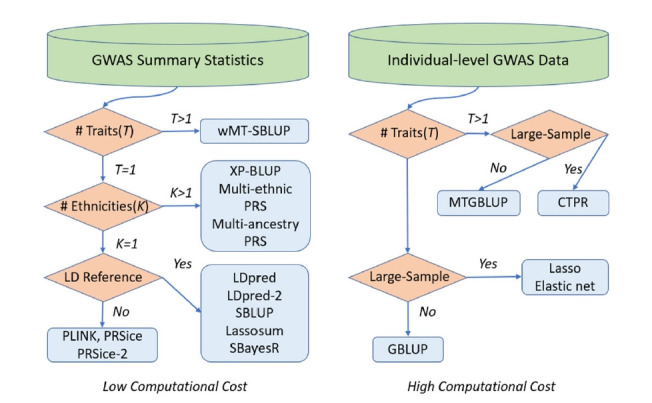
Best statistical models and software based on data type, sample size, LD reference panel, and the number of traits and ethnicities. CTPR, cross-trait penalized regression; GBLUP, genomic BLUP; GWAS, genome-wide association studies; LD, linkage disequilibrium; MTGBLUP, multi-trait GBLUP; SBLUP, statistics BLUP; wMT-SBLUP, weighted multi-trait SBLUP.

We have reviewed statistical models and computational tools for PRS computation. We have demonstrated a variety of statistical models for genomic risk prediction using individual-level data and/or summary statistics and showed how to improve prediction accuracy with multiple traits and multiple populations. Furthermore, we have introduced recent computational tools to conduct PRS analyses based on the statistical models, and explained how to specify the parameters and how to execute the software in detail. We also summarized which statistical models and software are best for specific situations based on data type (GWAS summary statistics or individual-level GWAS data), sample size, the LD reference panel, the number of traits, and the number of ethnicities, as shown in Fig. 1. The summary-based PRS methods such as PLINK, LDpred, and SBLUP offer advantages in computational cost over PRS methods using individual-level data such as GBLUP and the lasso method. This is because the computation time of summary-based PRS methods does not increase with the number of individuals in the study. This advantage has motivated the recent development of various summary-based methods in conjunction with LD information, although PRS methods using individual-level data could generate more accurate PRS. With recent large-sample GWAS data, summary-based methods are generally utilized due to their computational efficiencies, while PRS methods using individual-level data are still usable for computing more accurate PRS.

Despite the existence of various PRS methods, there are some areas in which further research on PRS is required. To improve prediction accuracy, we need novel statistical models and software that leverage information from multiple disease outcomes and multiple ethnicities based on individual-level genotype data and/or summary statistics from large-scale biobanks. It is also necessary to develop methods with the ability to predict diverse disease traits, such as cardiovascular disease and type 2 diabetes, with sufficient accuracy (to the extent allowable by disease heritability), and then these models need to be extended to utilize multiple ethnicities by incorporating information on LD to further improve prediction accuracy.

Moreover, with advances in high-throughput molecular assays (e.g., RNA-seq and ChIP-seq), it has been shown that disease risk SNPs are enriched in a broad array of functional regions, including regulatory features that are often tissue-specific, providing a novel source of information for improved prediction accuracy. It has been further shown that these molecular features can be predicted from genetic variants, enabling the prediction of gene expression in GWAS cohorts to perform transcriptome-wide association studies and to identify putative susceptibility genes. The accurate prediction of individual molecular features is now an emerging tool for discovering novel disease loci and characterizing biological mechanisms at the thousands of GWAS loci that have already been published. Data collection efforts of an unprecedented scale are now being seen in the areas of functional genomics and disease genetics. Such datasets can help to prioritize causal features and further improve prediction accuracy.

We conclude by emphasizing the importance of creating accurate PRS for a wide range of complex traits and diseases. The PRS provides an estimate of genetic predisposition (also called genetic susceptibility) for a complex trait or disease at the individual level, which refers to the likelihood of developing a particular trait or disease based on a genotype profile. The goal of PRS analysis is to identify individuals at an elevated risk of diseases on the basis of genetic variants in combination with clinical covariates. Therefore, the more accurate PRS we obtain, the better we can identify disease risk and the better we can provide treatment and prevention strategies. Personalized medicine based on accurate PRS will have a considerable impact on the treatment process and quality of life in the near future.

## Figures and Tables

**Table 1.** List of PRS methods, underlying statistical models, computational tools, and required data

Trait/Ethnicity	Method	Statistical model	Computational tool	Required data
Single trait, single ethnicity	PRS	Linear model	PLINK, PRSice, PRSice-2	Summary data
	LDpred	Bayesian model	LDpred, LDpred-2	Summary data
	GBLUP	LMM	GCTA	Individual data
	SBLUP	LMM	GCTA	Summary data
	BayesR	Bayesian model	GCTB	Individual data
	SBayesR	Bayesian model	GCTB	Summary data
	Penalized Regression	Penalized regression	glmnet	Individual data
	Lassosum	Penalized regression	lassosum	Summary data
Multiple traits, single ethnicity	MTGBLUP	Multivariate LMM	MTG	Individual data
	wMT-SBLUP	Multivariate LMM	wMT-SBLUP	Summary data
	CTPR	Multivariate penalized regression	CTPR	Individual data
Single trait, multiple ethnicities	XP-BLUP	Two-component LMM	XP-BLUP	Individual data
	Multi-ethnic PRS	Linear mixture approaches	multi-ethnic PRS	Summary data
	Multi-ancestry PRS	Linear mixture approaches	multi-ancestry PRS	Summary data

PRS, polygenic risk score; GBLUP, genomic BLUP; LMM, linear mixed model; GCTA, Genome-wide Complex Trait Analysis; SBLUP, statistics BLUP; GCTB, Genome-wide Complex Trait Bayesian Analysis; MTGBLUP, multi-trait GBLUP; wMT-SBLUP, weighted multi-trait SBLUP; CTPR, cross-trait penalized regression.
